# High Critical Current Density in the Textured Nanofiber Structure in Multifilament MgB_2_ Wires Made by the Powder-In-Tube (PIT) Technique

**DOI:** 10.3390/ma15155419

**Published:** 2022-08-05

**Authors:** Daniel Gajda, Andrzej J. Zaleski, Andrzej J. Morawski, Małgorzata Małecka, Lan Maria Tran, Matt Rindfleisch, Tomasz Durejko, Tomasz Czujko

**Affiliations:** 1 Institute of Low Temperature and Structure Research PAS, Okolna 2, 50-422 Wroclaw, Poland; 2 Institute of High Pressure Physics PAS, Sokolowska 29/37, 01-142 Warsaw, Poland; 3 Hyper Tech Research, Inc., 1275 Kinnear Road, Columbus, OH 43212, USA; 4 Institute of Materials Science and Engineering, Military University of Technology, Kaliskiego 2, 00-908 Warsaw, Poland

**Keywords:** textured nanofiber, MgB_2_ wires, high critical current density, powder-in-tube method

## Abstract

We show that the structure of multifilament MgB_2_ wires made by the powder-in-tube (PIT) method can be texturized by annealing the structure under high isostatic pressure. Our results show that we obtained continuous fibers with a uniform diameter of 250 nm in all 36 filaments, a small grain size of approximately 50 nm and a high density of the superconducting material. These results contribute to a significant improvement in the critical current density in high magnetic fields, e.g., 100 A/mm^2^ at 14 T and 4.2 K.

## 1. Introduction

The formation of the textured structure in superconducting wires and tapes is important because it allows a significant increase in the critical parameters, e.g., transport critical current density (*J*_c_) and irreversible (*B*_irr_) as well as upper (*B*_c2_) magnetic fields. Uchiyama et al. [1] showed that cold rolling a square wire using a two-axial grooved roller could create a textured fiber structure with a fiber diameter of 15 µm for a 1-mm diameter wire before annealing. However, annealing above 630 °C would lead to the disappearance of the textured fiber structure, and a large number of sizable voids would appear [1]. Moreover, Susner et al. [2] showed that the cold drawing process would lead to the elongation of Mg grains and a reduction in their thickness, and hence, structure texturization in the direction of the cold drawing axis was observed. Unfortunately, the texture structure deteriorated as a result of annealing even at a low temperature of 600 °C [2]. Beilin et al. showed that rolling and thermal treatment of MgB_2_ wires made by the PIT method poorly texturized the structure of the superconducting material [3].

It is well known that the textured structure in MgB_2_ materials after heat treatment can be obtained in thin layers. Currently, thin layers are formed by several methods, e.g., annealing of B films in Mg vapor [4], physical vapor deposition (PVD) [5] and hybrid physical–chemical vapor deposition (HPCVD) [6]. However, MgB_2_ materials have several features that hinder the formation of thin layers, e.g., Mg volatility, MgB_2_ phase stability, low Mg sticking rates at high temperatures, Mg reactivity to oxygen and carbon contamination [7]. The thin MgB_2_ layers are characterized by high critical parameters, e.g., high *B*_irr_ (approximately 37 T) and *B*_c2_ (approximately 45 T) [8]. However, this method for obtaining the textured structure is more expensive and complicated to apply than PIT methods.

Currently, the most common single- and multifilament MgB_2_ wires are made by the PIT method [9]. This technique is simple to perform and does not require the use of complicated and expensive equipment. The PIT method creates a significant reduction in the production cost of MgB_2_ wires. The disadvantages of in situ MgB_2_ wires made using the PIT technique are the inhomogeneous structure and the low density of the superconducting material after thermal [2].

Our previous studies showed that annealing under high isostatic pressure using the hot isostatic pressing (HIP) process enhanced the textured (layered) structure in MgB_2_ wires made by the PIT method, e.g., more layers, a smaller layer thickness of approximately 25 µm and a higher density of the superconducting material [10]. Additionally, the HIP process produced a significant increase in *J*_c_ in MgB_2_ wires made by the PIT method. Moreover, the HIP process created structural defects, e.g., dislocations that acted as pinning centers [11].

In this paper, we present the opportunity to manufacture textured long multifilament MgB_2_ wires made using the PIT method and subsequent annealing under high isostatic pressure. The structure of the obtained wires is characterized by the presence of fibers with a uniform diameter of 250 nm, small grains of 50 nm, exceptional connections between the grains and extremely high *J*_c_ (100 A/mm^2^ in 14 T at 4.2 K).

## 2. Materials and Methods

The 36-filament MgB_2_ wire in the Nb barrier was manufactured using a continuous tube forming and filling (CTFF) process [9]. The fibers were produced from a mixture of boron nanopowder pre-doped with 2 at. % C, and magnesium with a Mg-to-B ratio of 1:2. The wires were pulled to a diameter of 0.83 mm, achieving a fill factor of 14%. Samples A and B were annealed at 700 °C for 15 min under low (0.1 MPa) and high (1 GPa) isostatic pressures, respectively [12,13]. The transport critical current (*I*_c_) of the MgB_2_ wires was measured by the four-probe resistive method at 4.2 K [13,14]. The *I*_c_ was determined on the basis of a 1 V/cm criterion. The critical current density (*J*_c_) was determined from the relationship *J*_c_ = *I*_c_/S where S is the surface of the superconducting material. The critical temperature (*T*_c_) and the critical magnetic fields (*B*_irr_ and *B*_c2_) were measured using the four-probe resistive method on a physical properties measurement system (PPMS). The *T*_c_, *B*_irr_, and *B*_c2_ were determined with the respective criteria of 50%, 10%, and 90% of the normal state resistance. Transport measurements were performed with the measurement error ranging from 2% to 4%. Analysis of the microstructure and composition was performed using scanning electron microscopy SEM; FEI Nova Nano SEM 230 (Hillsboro, OR, USA).

## 3. Results and Discussion

**Figure 1:**
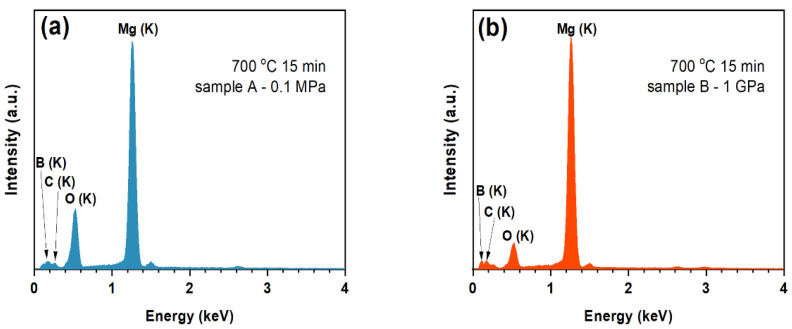
The EDS analysis of samples longitudinal-section for (**a**) sample A—0.1 MPa and (**b**) sample B—1 GPa.

**Figure 2:**
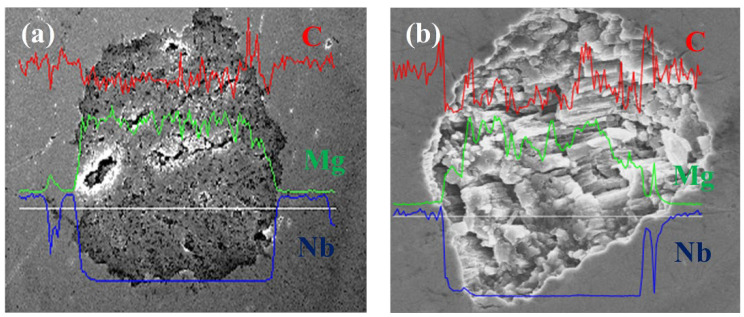
Linear analysis of sample composition for the cross-section (**a**) of sample A—0.1 MPa and (**b**) sample B—1 GPa. The red color means carbon (C), green—magnesium (Mg), dark blue—niobium barrier (Nb).

The energy dispersive X-ray spectroscopy (EDX) studies (Figure 1) and the linear composition analyses (Figure 2) of the longitudinal and transverse sections indicated that the superconducting material in samples A and B had high purity and the components had a homogeneous distribution. These results indicate that the Nb barrier provides strong protection for the MgB_2_ material against contamination. Additional components (e.g., oxygen (O)) appear in the structure of sample during the preparation for analysis by using scanning electron microscopy (SEM). Moreover, the quality of the Nb barrier was checked by using the transport method—temperature sweep [15].

**Figure 3:**
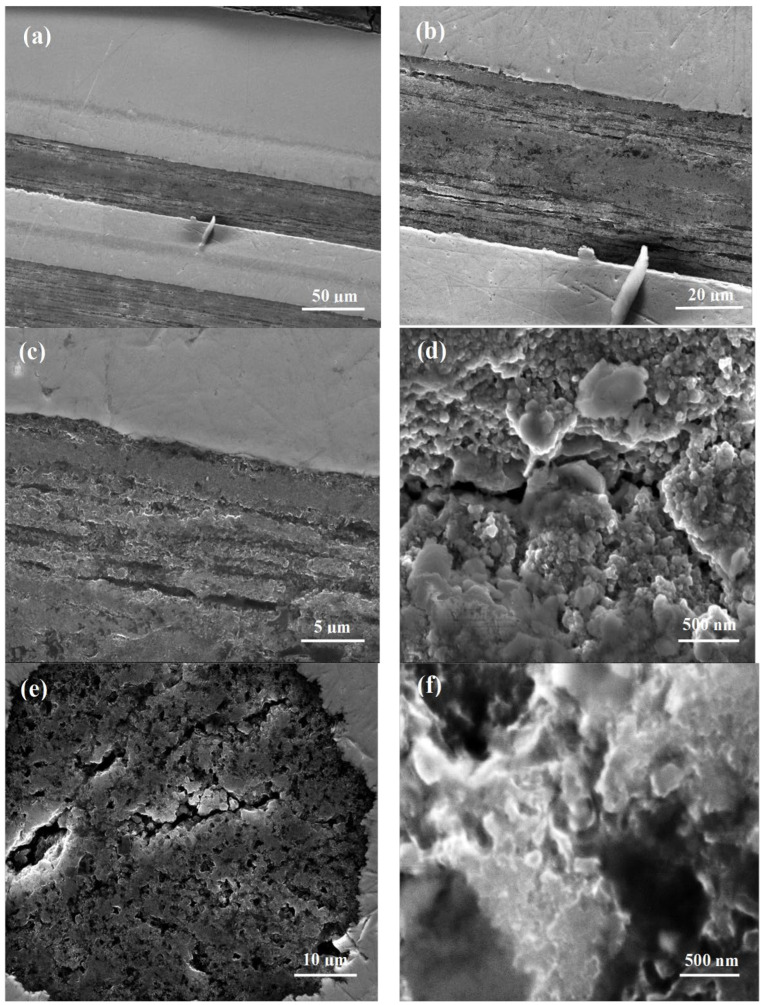
SEM photos (**a**–**d**) longitudinal-sections and (**e**,**f**) cross-sections for sample A annealed at 700 °C under isostatic pressure of 0.1 MPa for 15 min.

The low magnification SEM photos (longitudinal section in Figure 3a show that the structure of the superconducting material was similar in all the filaments of sample A, which were annealed under isostatic pressure of 0.1 MPa. Further results in Figure 3b,c indicate that sample A had a layered structure with a layer thickness ranging from 1 µm to 20 µm, long void lengths over 50 µm and a width of up to 1 µm. Moreover, the results in Figure 3a–c show a discontinuity in the layered structure. This discontinuity reduced the number of connections between layers and intergrain connections. The large magnification of the longitudinal section in Figure 3d shows that sample A had a grain size between 50 nm and 250 nm. Additionally, Figure 3d shows that the grains grew in both the longitudinal and transverse directions. The results for the low-magnification cross-section show that sample A had a large number of voids that reached 10 µm in size (Figure 3e). High magnification SEM images of the cross-section indicate that sample A had grain sizes ranging from 50 nm to 200 nm and void sizes of 500 nm.

**Figure 4:**
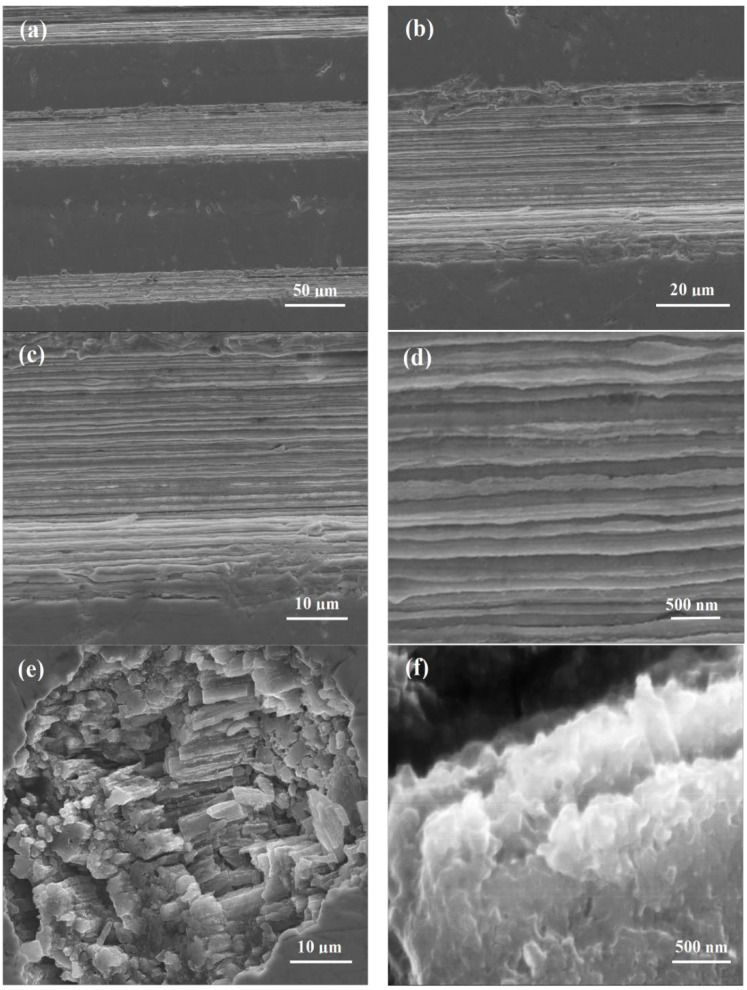
SEM photos (**a**–**d**) longitudinal-sections and (**e**,**f**) cross-sections for sample B annealed at 700 °C under isostatic pressure of 1.0 GPa for 15 min.

The studies performed for sample B (longitudinal section) show that the structure of the superconducting material is very similar in all filaments (Figure 4a–c). This indicates that the superconducting material has a layered structure, no voids, a large density of the superconducting material and the same size and shape of each layer. Further SEM studies displayed in Figure 4d show that the layers were approximately 250 nm thick and grew mainly in the longitudinal direction. The growth in the transverse direction was negligible. These results indicate that sample B had a textured structure in the direction of the cold drawing axis. The SEM images of the cross-section for sample B (Figure 4e,f) show that the grains were 50 nm in size and grew mainly in the longitudinal direction, and only a few voids were visible, which implies that the MgB_2_ material has a high density. The results in Figure 4 show that sample B had a very large number of connections between the layers and the grains. By comparing the results in Figure 3 with Figure 4, we can see that the HIP process significantly increased the homogeneity and density of the MgB_2_ material, significantly reduced the grain size, created thin, uniform layers and increased the uniformity of the MgB_2_ material.

Uchiyama et al. [1] and Susner et al. [2] indicated that cold work textures the structure of MgB_2_ wires made by the PIT method and reduces the thickness of Mg grains. In our work, the small Mg grains grew faster in the longitudinal and transverse directions (Figure 3d,f) than the large Mg grains [2]. This trend made it difficult to maintain a regular textured structure after annealing. Earlier studies showed that the fibers in the textured structure were 15 µm and 25 µm in size [1,10]. In sample B, we obtained fibers with a textured structure two orders of magnitude smaller (250 nm). Moreover, the fiber thickness was similar to the thickness of thin MgB_2_ layers (150 nm) obtained by the HPCVD method [6]. Furthermore, in sample B, we obtained MgB_2_ grains similar in size to MgB_2_ grains in thin layers (40 nm) [1].

**Figure 5:**
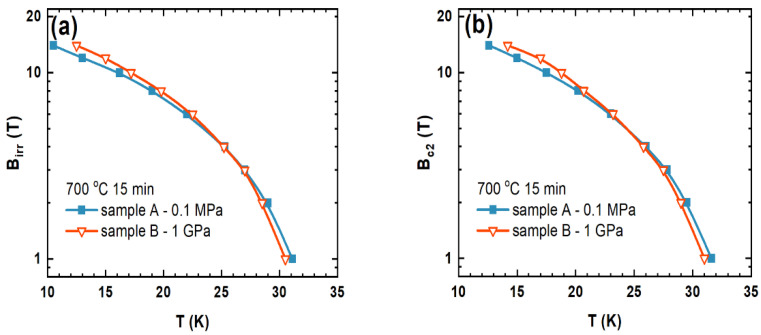
Transport measurements: (**a**) temperature (*T*) dependence on the irreversible magnetic field (*B*_irr_) and (**b**) temperature (*T*) dependence on the upper critical field (*B*_c2_).

**Figure 6:**
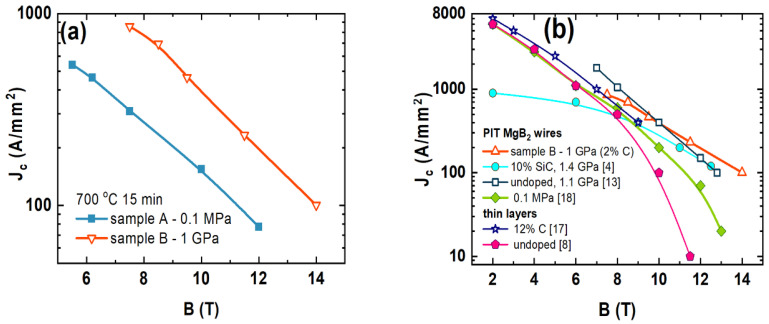
(**a**) Dependence of the perpendicular magnetic field (*B*) on the transport critical current density (*J*_c_) at 4.2 K for samples A and B and (**b**) for comparison, the results of undoped and doped MgB_2_ wires made by using PIT method and thin layers, e.g., C-doping and SiC-doping.

The transport measurements showed that sample B had a *T*_c_ that was 1.5 K lower than sample A. The reduction in *T*_c_ was caused by the structural defects that the HIP process created [16]. In Figure 5, we can observe that sample B had slightly higher *B*_irr_ and *B*_c2_ in the temperature range from 10 K to 25 K than sample A [17]. However, above 25 K, sample A had slightly higher *B*_irr_ and *B*_c2_ than sample B [17]. The values of *B*_irr_ and *B*_c2_ depended on the pinning centers [17]. Our results show that the HIP process allows to improve and increase the density of pinning centers in the range of low and middle temperatures, e.g., dislocations [16]. Moreover, the results in Figure 5 indicate that the HIP process creates weaker pinning centers at high temperature. Our results might suggest that dislocations trap the vortex lattice more efficiently at low and middle temperatures than at high temperatures. This observation indicates that the HIP process slightly affected the dominant pinning mechanism. Our samples have *B*_irr_ and *B*_c2_ values similar to the *B*_irr_ and *B*_c2_ values of the thin layer, e.g., sample B had the *B*_irr_ of 4 T at 24 K and a thin layer of 500 nm had the *B*_irr_ of 5 T at 25 K [8]. The results in Figure 6a show that sample B had a significantly higher critical current density (three times greater) than sample A. Sample B had the *J*_c_ of 100 A/mm^2^ in the perpendicular magnetic field with a magnetic flux density of 14 T. The textured structure and the HIP process created a large number of connections between the grains and layers and allowed a large number of pinning centers to be obtained.

The results in Figure 6b show that sample B had a much higher *J*_c_ in high magnetic fields than thin MgB_2_ layers [8,18] or PIT MgB_2_ wires annealed under low [19] and a pressure of 1.4 GPa [13,14]. This result indicates that the textured structure that appears in multifilament PIT MgB_2_ wires with small grains and nanofibers along with the HIP process allows for the creation of more connections and a high density of high-field pinning centers, e.g., dislocations, strains, and substitutions to the crystal lattice. Our results show that the method to obtain the aforementioned textured structure is the only technique that can produce very high *J*_c_ in the high magnetic field in PIT MgB_2_ wires.

## 4. Conclusions

The results show that heat treatment under a high isostatic pressure of 1 GPa allows us to obtain a textured structure with a high density of superconducting material, a uniformity and homogeneity of layers in each filament, a layer thickness of 250 nm, a grain size of 50 nm and no voids. Additional studies showed that in sample B, after the HIP process, the structure grew mainly in the longitudinal direction. On the other hand, in the sample annealed at the low isostatic pressure of 0.1 MPa, the structure grew in the longitudinal and transverse directions. Our research shows that the textured structure and HIP process can obtain the highest *J*_c_ in MgB_2_ wires made by the PIT method in high magnetic fields.

## Data Availability

The data presented in this study are available on reasonable request from the corresponding authors.
